# Riok1, A Novel Potential Target in MSI-High p53 Mutant Colorectal Cancer Cells

**DOI:** 10.3390/molecules28114452

**Published:** 2023-05-31

**Authors:** Sharon Shechter, Sapir Ya’ar Bar, Hamdan Khattib, Matthew J. Gage, Dorit Avni

**Affiliations:** 1 Department of Chemistry, University of Massachusetts Lowell, Lowell, MA 01854-2874, USA; sharon_shechter@student.uml.edu (S.S.); matthew_gage@uml.edu (M.J.G.); 2 Department of Natural Compound, Nutrition, and Health, MIGAL Galilee Research Institute, Kiryat Shmona 1101600, Israel; sapiry@migal.org.il (S.Y.B.); hamdanm@migal.org.il (H.K.)

**Keywords:** microsatellite instability (MS), colorectal cancer cells (CRC), p53, KRAS, synthetic lethality, kinase, kinase-related pathways

## Abstract

The vulnerabilities of cancer cells constitute a promising strategy for drug therapeutics. This paper integrates proteomics, bioinformatics, and cell genotype together with in vitro cell proliferation assays to identify key biological processes and potential novel kinases that could account, at least in part, for the clinical differences observed in colorectal cancer (CRC) patients. This study started by focusing on CRC cell lines stratified by their microsatellite (*MS*) state and p53 genotype. It shows that cell-cycle checkpoint, metabolism of proteins and RNA, signal transduction, and WNT signaling processes are significantly more active in MSI-High p53-WT cell lines. Conversely, MSI-High cell lines with a mutant (Mut) p53 gene showed hyperactivation of cell signaling, DNA repair, and immune-system processes. Several kinases were linked to these phenotypes, from which RIOK1 was selected for additional exploration. We also included the KRAS genotype in our analysis. Our results showed that RIOK1’s inhibition in CRC MSI-High cell lines was dependent on both the p53 and KRAS genotypes. Explicitly, Nintedanib showed relatively low cytotoxicity in MSI-High with both mutant p53 and KRAS (HCT-15) but no inhibition in p53 and KRAS WT (SW48) MSI-High cells. This trend was flipped in CRC MSI-High bearing opposite p53-KRAS genotypes (e.g., p53-Mut KRAS-WT or p53-WT KRAS-Mut), where observed cytotoxicity was more extensive compared to the p53-KRAS WT-WT or Mut-Mut cells, with HCT 116 (KRAS-Mut and p53-WT) being the most sensitive to RIOK1 inhibition. These results highlight the potential of our in silico computational approach to identify novel kinases in CRC sub-MSI-High populations as well as the importance of clinical genomics in determining drug potency.

## 1. Introduction

Colorectal cancer (CRC) is the third-most-common cancer in Western countries and the second leading cause of cancer-associated death globally [1]. CRC shows a significant heterogeneity in both prognosis and response to therapy, which may be linked to genetic alterations that occur during pathogenesis. In 85% of CRCs, disease progression is driven by chromosomal instability (CIN), while the defective function of the DNA mismatch repair (MMR) system is responsible for the other 15% of CRCs, resulting in a phenotype called microsatellite instability (MSI) [2]. The normal MMR system is organized around a complex of MSH (MSH2, MSH3, and MSH6) and MLH (MLH1, PMS1, PMS2) proteins that signal repair of the base–base mismatches and short insertion/deletion mispairings that spontaneously occur during DNA replication [3,4].

Microsatellites (MS) are short DNA tandem repeats (1–6 nucleotides) that represent approximately 3% of the human genome. MS generation is believed to occur by DNA slippage during DNA replication and repair, resulting in repeating nucleotides being skipped or inserted. Microsatellite instability (MSI) occurs when mismatch repair (MMR) enzymes are inactive and tumors containing at least two of the five known MSI markers are referred to as MSI-High cell lines [5]. In CRC, MSI-induced frameshift mutations have been identified in genes associated with a variety of cellular functions, including DNA repair (MSH3 and MSH6), cell signaling (TGFBR2 and ACVR2A), apoptosis (BAX), epigenetic regulation (HDAC2 and ARID1A), and miRNA processing (TARBP2 and XPO5) [6]. Cell lines without a high percentage of MS events are considered microsatellite-stable (MSS) cell lines. In CRC patients, 15% are characterized as MSI whereas the remainder are MSS [7]. There are two known MSI states: high microsatellite instability (MSI-High), and low microsatellite instability (MSI-Low). At present, clinical research tends to classify MSI-Low and MSS as one as MSI-Low colorectal tumors have not been shown to differ in their clinicopathologic features or most molecular features from MSS tumors [8,9]. 

Genomic-wise, CRC cell lines possess a high frequency of TP53 (60%) mutations. Dysregulation of the p53 tumor suppressor gene is one of the most frequent factors controlling the aggressive and metastatic features of CRC. Half of all colorectal cancers containing p53 mutations, appear to be more chemo-resistant and have a poorer prognosis than those with wildtype (WT) p53 [10,11,12]. In addition, CRC cell lines also possess KRAS (49%) mutations. In detail, *KRAS*-mutated CRCs had a lower frequency in MSI-High (15% vs. 42%; *p* = 0.015) compared with KRAS-WT, though KRAS mutation was associated with higher tumor-cell turnover [13].

A study of Bosnian colorectal cancer patients identified a potential link between alterations in certain tumor-suppressor genes and the MSI phenotype [14]. Specifically, it was noted that 75% of colon cancers show a loss of heterozygosity (LOH) on chromosomes 17p, 18q, and 5q where tumor suppressor genes such as APC, MCC, DCC, and p53 are known to be located [15]. Our in silico study focused on CRC MSI-High cells stratified by their p53 genotype. We postulated that it might be possible to identify a novel kinase as a synthetic lethality (SL) partner for CRC MSI-High cell lines. Synthetic lethality arises when a combination of deficiencies in two or more genes arises from mutations, epigenetic alterations, or inhibition and leads to cell death that does not occur when only one gene is defective. Support for this hypothesis comes from studies that demonstrated that CSNK1E is a promising druggable target for a broad population of TP53-mutant (Mut) CRC patients [16]. Although CRC bears a high frequency of TP53 (60%) and KRAS (49%) mutation, a recent publication indicated that these two are mutually exclusive in KRAS CRC-altered tumors [17]. To provide the most effective therapeutic approach to the indicated subpopulation, in our in silico analysis we focused on p53 alterations while ‘tracking’ the KRAS genotype. 

This work integrates cell genotype, proteomics, bioinformatics, and in vitro cell proliferation studies to identify key biological processes and kinases that could account for the clinical differences observed between CRC patients. In silico analysis was done on 34 CRC cell lines stratified by their MS status (MSI and MSS) and p53 (wildtype and mutant) genotype with a focus on differences in pathways activation levels and kinase abundance. We hypothesized that targeting highly abundant kinases in MSI-High cell lines, which have specific vulnerabilities due to inactive MMR genes, might disrupt the delicate balance between cell proliferation and cell death, inducing apoptosis. The rationale for this approach is three-fold. First, some mutant p53 proteins lose their tumor-suppressor activity and gain oncogenic functions, providing advantages to survival [18]. Second, kinases are ideal partners for SL because dynamic phosphorylation plays a major role in cancer initiation, progress, and drug response [19]. Finally, MSI-High CRCs are characterized by clinical and pathologic features distinct from those observed in MSS and patients with MSI-High show limited benefit from Fluorouracil (5FU)-based adjuvant therapy, while 5FU has a curative effect on patients with advanced stages CRC [20,21]. This supports the urgent need to identify a novel kinase as an alternative treatment [22].

To address this need, we investigated whether a novel SL partner could be identified in CRC cells. There are known differences in the activity level of signaling proteins in MSI-High p53-WT and MSI-High p53-Mut cell lines, and gene expression is altered in CRC cells with MSI compared to non-MSI [23]. In our analysis, cell-cycle checkpoint, metabolism of proteins and RNA, signal transduction, and WNT signaling process were significantly more active in MSI-High p53-WT cell lines. In contrast, cell signaling, DNA repair, and immune system processes were significantly more active in MSI-High p53-Mut cell lines. There were also differences in kinase abundance between the MSI-High subgroups from which RIOK1 was selected for further investigation. We decided to focus on RIOK1 because it seemed to closely relate to both TP53 and KRAS-mutated CRC. RIOK1 was highly abundant in CRC MSI-High cell lines and CRISPR KO of RIOK1 showed dependency in CRC MSI-High cell lines and Weinberg et al. documented an increased dependency of RIOK1 in tumor cells with oncogenic Ras signaling [24]. Based on these facts, we hypothesized that RIOK1 could serve as a great SL partner in the context of sub-MSI-High CRC cell lines. 

We tested CRC MSI-High (both p53/KRAS and WT/Mut) cell lines using Nintedanib, a known RIOK1 inhibitor, to validate this hypothesis. As a control, 5FU was used as it is a first-line treatment for colorectal cancer [25,26]. Our in vitro results demonstrated inhibition of CRC MSI-High cell proliferation in a p53 and KRAS genotype-dependent manner when treated with 5FU or Nintedanib. In SW48, p53, and KRAS WT cell line, there was complete resistance to Nintedanib, while in HCT 116 and LS411N, p53, and KRAS with the opposite genotype (one WT and the other is Mut) showed significant cytotoxicity (89% and 52%, respectively). Interestingly, HCT-15 (p53 and KRAS double Mut cell) showed low cytotoxicity (37%) when treated with Nintedanib. Taken together, the present study suggests a new mechanism for identifying novel kinases to identify potential new targets tailored toward a sub-MSI-High CRC population. Our findings suggest that RIOK1 might be a potential partner for the synthetic lethality mechanism involving p53 and KRAS, though further research is required to validate this possibility. 

## 2. Results

Mantovani et al. have suggested that mutant p53 proteins can favor cancer-cell survival and tumor progression by acting as homeostatic factors that sense and protect cancer cells from transformation-related stress stimuli, including DNA lesions, oxidative and proteotoxic stress, metabolic imbalance, interaction with the tumor microenvironment, and the immune system [27]. Similarly, *KRAS* mutations confer the constitutive activation of this oncogene, stimulating cell proliferation, inducing autophagy, suppressing apoptosis, altering cell metabolism, changing cell motility and invasion, and modulating the tumor microenvironment [28]. These mutants’ activities may disclose tumor vulnerabilities and synthetic lethality that could be exploited by targeting tumors bearing either TP53 or KRAS mutations [27]. Our initial hypothesis focused on p53 mutations while ‘tracking’ the KRAS genotype as an SL partner due to its very high prevalence in MSI-High CRC cell lines.

CRC cell lines can be divided based on MS status and p53 genotype: Colorectal tumors with MSI have distinctive features, including a tendency to arise in the proximal colon, lymphocytic infiltrate, and a poorly differentiated, mucinous, or signet ring appearance. While they have a slightly better prognosis with immune therapy than MSS colorectal tumors, they do not show improved survival in CRC stages II and III compared to MSI-High patients treated with chemotherapy [23]. Based on these factors, we investigated the differences in key pathways and essential proteins between CRC cell lines with differences in either MS status, p53 genotype, or a combination of the two. We combined data regarding the p53 genotype and MS status in CRC cell lines since MS status was not unanimous across the different databases [26,29]. Based on all these criteria, a final list of eight MSI-High cell lines was identified, of which three had a p53 mutant genotype (LS411, SNU-C2B, and CCK-81), and five had a wild-type p53 (WT) genotype (LS180, HCT 116, RKO, LoVo, and SW48). 

**Figure 1:**
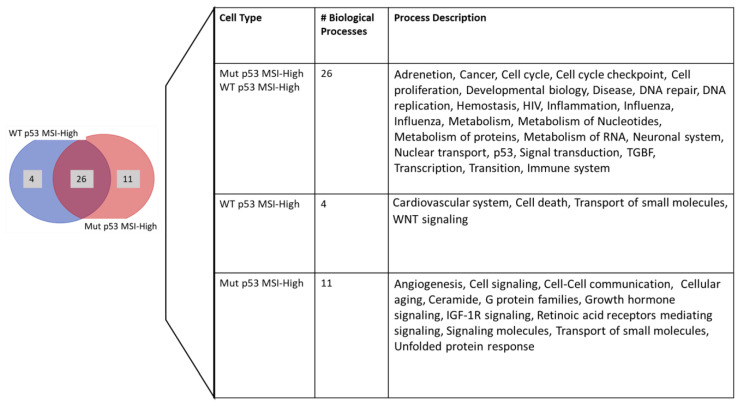
Highly activated biological processes found in MSI-High cell lines stratified by their p53 genotype Highly activated pathways were clustered into 41 biological processes of which 26 were highly activated in both MSI-High p53 Mut and WT; 4 processes are unique to MSI-High p53-WT and 11 to MSI-High p53-Mut.

Unique highly activated pathways exist in MSI-High p53-Mut cell lines: The pathways that are most active in MSI-High cell lines were then investigated using the ATLANTiC portal [29]. Pathways were considered active in a particular cell line when their reported relative activity score was above 0.85, as this threshold has been utilized in other studies [29]. To further refine our search, we focused on kinases that may account for those pathways’ activation levels in CRC MSI-High cell lines based on the p53 genotype. This resulted in the identification of 325 highly active pathways in the MSI-High p53-Mut cell lines and 485 pathways in the MSI-High p53-WT cell lines. Related pathways were then clustered based on annotation from the Gene Set Enrichment Analysis database (GSEA DB) [30,31,32,33,34,35,36,37,38,39], PID [40], and BioCarta [41] databases. Each of the databases used for clustering tends to use different terms to describe similar concepts. For example, KEGG uses more broad terms while Reactome uses similar terms but as multiple detailed entries (split terms for same entry from KEGG). This can explain the appearance of both “cell signaling” and “WNT signaling” (which is a subcategory under cell signaling) in the pathway analysis. This resulted in an overall dataset where 26 processes appeared to be common to both the p53-WT and p53-Mut MSI-High cell lines while 4 appeared in only p53-WT and 11 in only the p53-Mut cell line (Figure 1). 

Further examination showed that eight specific processes were found to have statistically significantly different (*p* < 0.01 or *p* < 0.05) levels of activation between the MSI-High p53-WT and MSI-High p53-Mut using a chi-squared analysis (Table 1).

Two general trends were apparent within these eight processes. First, cell signaling, DNA repair, and immune-system-related processes were significantly more activated in MSI-High p53-Mut cell lines while cell-cycle checkpoint, metabolism of protein, metabolism of RNA, signal transduction, and WNT signaling were more pronounced in MSI-High p53-WT cell lines. These observations are consistent with previous studies that have shown metabolism-related pathways are enriched in MSI-High p53-WT cells and the presence of p53 is required for tumor cells to adapt to the metabolic changes necessary to tolerate drugs that induce metabolic stress [39,40]. Second, immune-system processes were activated in both MSI-High p53 subgroups but they were significantly more activated in MSI-High p53-Mut. This observation is consistent with studies that showed that high mutational load in MSI tumors leads to tumor-specific neoantigens [42,43]. A correlation between mutation of p53 and p53 antibodies has been previously shown in ovarian, lung, colorectal, breast, and liver cancer patients, providing a potential explanation for the significantly higher level of immune system processes observed in MSI-High p53-Mut cell lines [44]. Other studies have shown that mutant p53 in cancer cells can be effectively processed and presented by the major histocompatibility complex (MHC), causing an immunogenic reaction [45,46]. This leads to a cancer-specific immune response that could explain the response of p53-Mut epithelial cancers to immunotherapy [22,46,47]. Though all MSI-High cell lines are known to have inactivated MMR genes and altered DNA repair mechanisms; MSI-High p53-Mut cell lines showed further hyperactivation of immune system-related processes, matching recent results suggesting a wide array of neomorphic activities are contributing to cancer progression due to missense p53 mutants [27]. Though it may not be possible to infer sensitivity toward a specific drug due to differentially regulated pathways, these results could highlight important kinases that could be potential SL partners when highly abundant. This could result in a new anticancer therapy with reduced/no target toxicity for a specific subpopulation. 

TBK1, RIOK1, CAMK1, and CHUK are upregulated in MSI-High cell lines: Having identified biological processes that are activated differently between MSI-High p53-WT and MSI-High p53-Mut, we wanted to identify the most abundant kinases that could explain these differences. Using the ATLANTiC’S portal [29] and a threshold level of >0.85, four enriched kinases were identified in MSI-High cell lines: TBK1 (foreign DNA and RNA/immune response signaling), RIOK1 (ribosome biogenesis in eukaryotes), CAMK1 (a component of a calmodulin-dependent protein kinase cascade), and CHUK (NF-Kβ signaling) (Table 2). We also analyzed fourteen MSS cell lines, of which four had a mutant p53 and ten had a WT p53, and determined that MSI-High cell lines have a different pattern of active biological processes and kinases compared with the MSS cell lines (Table S1). This is consistent with the hypothesis that CRC MSI-High cell lines exhibit clinical, pathological, and molecular characteristics that distinguish them from MSS cancer cell lines.

**Figure 2:**
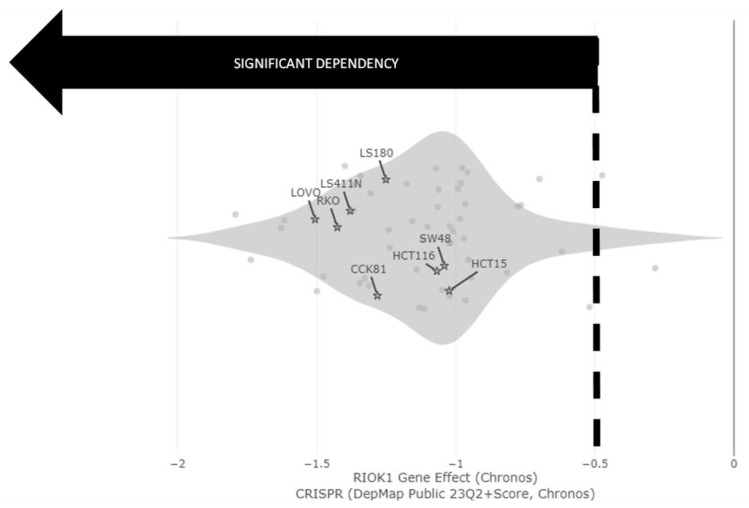
RIOK1 CRISPR Knockout in CRC MSI-High cell lines: CRC MSI-High cell lines dependency on RIOK1 CRISPR KO. Cell lines are considered to be dependent when their gene effect (<−0.5). Labeled cells are those that were used in our study. Cell genotype: LS411N KRAS (WT) and p53 (Mut), CCK-81 KRAS (WT) and p53 (Mut), LS180 KRAS (G12D) and p53 (WT), HCT116 KRAS (G13D) and p53 (WT), HCT15 KRAS (G13D) and p53 (Mut), RKO KRAS (WT) p53 (WT), LoVo KRAS (G13D; V14A) p53 (WT), and SW48 KRAS (WT)) and p53 (WT).

The enrichment in the abundance of RIOK1 (ATLANTiC’s portal) (Table 2) is consistent with the data from the Cancer Dependency Map Portal (DepMap), showing higher expression levels of RIOK1 in MSI cell lines compared to MSS (Figure S1). RIOK1 dependency in MSI-High was further supported in CRISPR KO results using DepMap (Figure 2). This figure emphasized that all of the MSI-High cell lines tested were dependent on RIOK1 since their gene effect was reported to be (<−0.5).

Recent publications have demonstrated an SL role for RIOK1 in methylthioadenosine phosphorylase (MTAP)-depleted cells and its overexpression in colon cancer cells was shown to promote cell proliferation in vitro in human CRC [24,48]. However, the strongest rationale for selecting RIOK1 for further investigation had to do with the limited efficacy and intrinsic resistance seen in phase I/II clinical trials of patients treated with Sotorasib and Adagrasib, (KRASG12C selective inhibitors [49,50]) and the fact that knockdown of RIOK1 strongly impairs proliferation and invasiveness in conventional and 3D culture systems in RAS-mutant cancer cells [24]. These findings pointed towards a specific requirement for RIOK1 in the context of oncogenic RAS signaling that could overcome the observed clinical phenomenon. Thus, our hypothesis of RIOK1 acting as an SL partner in MSI-High cells bearing abnormal p53 coincides well with RIOK1′s role in RAS-mutated CRC cell lines. The prediction was that by blocking RIOK1, we could promote the cell susceptibility required for apoptosis and mark RIOK1 as a potential anticancer therapy, representing a new strategy for targeting CRC MSI-High p53-Mut cell lines.

**Figure 3:**
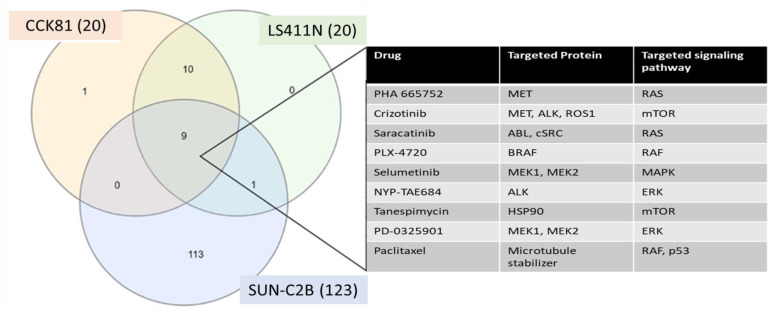
Venn diagram of known drugs potent against MSI-high p53 Mut cell lines. Known drugs that were noted to be potent in MSI-High Mut p53 cell lines. 9 drugs were shown to be common to all MSI-high p53 Mut cell lines targeting known cancer pathways: RAS-MAPK signaling pathway (MAPK- Raf-ERK-MEK) and mTOR.

Known Kinase inhibitor drugs and their downstream pathways in p53-Mut MSI-High cell lines: Using data published by Frenjo, we generated a Venn diagram (Figure 3) that depicts nine drugs known to be potent against MSI-High Mut p53 and their targeted pathways [29]. 

**Figure 4:**
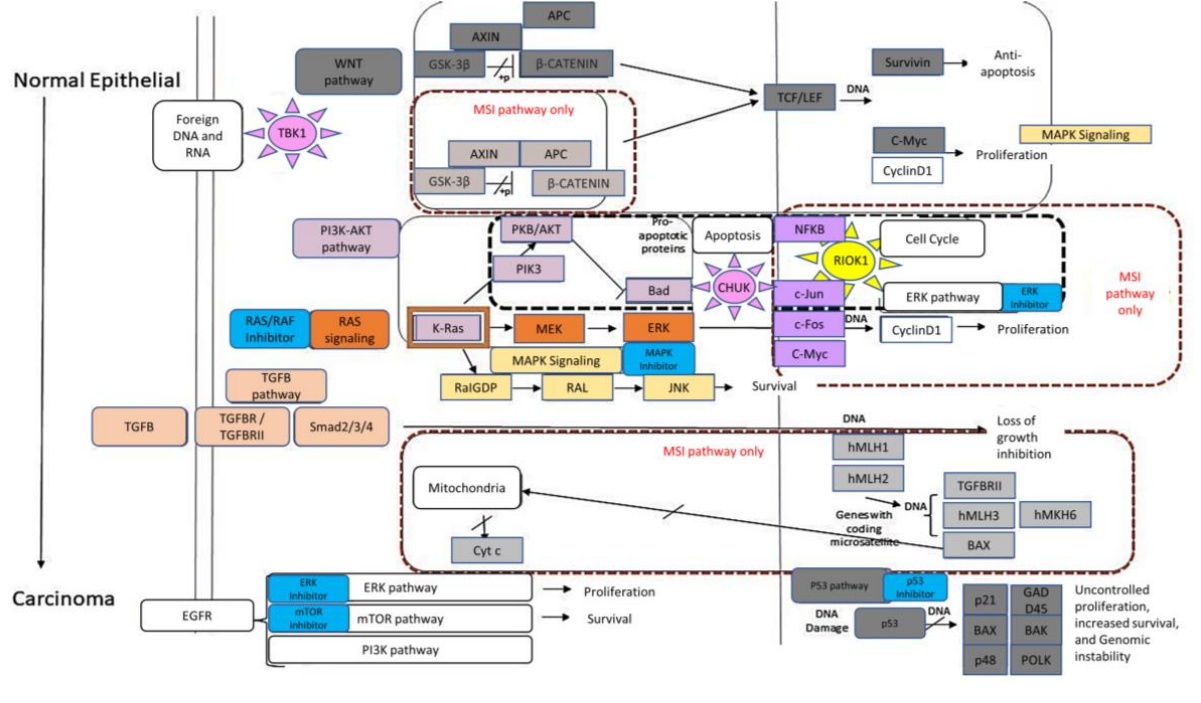
CIN and MSI signaling pathways in colorectal epithelial cancer: A modified cartoon from KEGG depicts known activated pathways in colorectal epithelial cells. Cyan rectangles are known anticancer-targeted genes. The pink\yellow sun symbols represent newly identified kinases in the context of MSI-high p53 Mut cell lines that could serve as an SL partner. The sun is located next to the pathway that it belongs to. MSI-only pathways are highlighted in red and MSI-high p53 Mut-specific pathways are in black.

Those nine drugs belong to six pathways (highlighted in cyan Figure 4) and are part of major signaling pathways known to be activated in the transformation of normal epithelial cells to carcinoma. Our goal was to identify a novel kinase whose inhibition is outside these known signaling pathways.

RIOK1 pathway inhibition for MSI-High CRC treatment: Pathways critical to the transformation of normal colorectal epithelial cells to carcinoma in CRC-MSI and CRC-MSS cell lines and MSI-High specific pathways are noted in Figure 4 [23]. Some examples of these pathways include transforming growth factor-β (TGFβ) signaling, which is known to inhibit proliferation in the colonic epithelium but is absent in MSI-High cells because TGFβ type II receptor (TGFβR2) is not expressed in these cell lines [51]. The phosphatidylinositol 3-kinase-AKT (PI3K) pathway acts as a ‘driver’ of tumorigenesis when treated with rapamycin, a PI3K inhibitor [52,53]. Finally, adenomatous polyposis coli (APC) is highly mutated in CRC and is part of the canonical WNT/wingless pathway that is aberrantly upregulated in MSI cell lines [54].

RIOK1′s role and its high abundance in MSI-High cell lines led to our hypothesis that inhibition of this kinase could induce apoptosis through an SL mechanism involving p53 or KRAS as partners. In addition, RIOK1 is required for AKT-, EGFR-, and PI3K-driven tumorigenesis, and reducing its activity could potentially be lethal to cells expressing pathological levels or mutant versions of these oncogenic factors [55,56,57]. Finally, RIOK1 plays an important role in ribosomal biogenesis and as a substrate recruiting protein of the protein arginine methyltransferase (PRMT5) complex, a promoter of CRC cell proliferation, migration, and invasion [24,58]. These downregulated proteins were associated with cell cycle or metabolic activity [58,59]. In addition, this paper suggested a hypothetical model where IL-1β stimulation activates the NF-κB pathway and induces PKCι-mediated phosphorylation of PRMT5, opening-up novel therapeutic avenues for CRC patients based on disrupting the PKCι/PRMT5/NF-κB and RIOK1 signaling axis [48]. Lastly, a recent publication showed that the drug levosimendan modulates RNA processing through inhibition of the RIOK1 pathway, consistent with our in silico biological process analysis showing a significant increase in the activity of this pathway in all MSI-High cell lines (Table 1). This model is supported by results showing that knocking down RIOK1 in RAS-dominant colon, breast, and lung cancer cells impaired their ability to proliferate and invade [24]. Genetic lethality screens in human cells have also shown that RIOK1 increases RAS GTPase activity, suggesting that functional RIOK1 is required for oncogenic gain-of-function RAS signaling. All of these studies highlight that RIOK1 may have great clinical relevance, even though it is relatively unstudied compared to other kinases [60,61,62]. To summarize, the depletion of RIOK1 affects the expression of several proteins involved in key biological processes e.g., ribosomal biogenesis, cell cycle progression, metabolic activity, and NF-κB signaling, all of which can justify its potential as the perfect partner for SL in MSI-High p53-Mut cell lines.

Data in the Achilles project database demonstrate that the “essentiality” for RIOK1 expression differs between the various CRC MSI-High cell lines in a CRISPR/Cas9 screen (https://portals.broadinstitute.org/achilles (accessed on 21 May 2023)), further supporting our hypothesis that some tumor cells are reliant on RIOK1 expression (Figure 2). A KEGG pathway map of the known CIN and MSI pathways in colorectal epithelial cells (https://www.genome.jp/pathway/map05210 (accessed on 2 September 2021)) was analyzed to visualize how inhibition of RIOK1 might trigger novel pathways in MSI-High cell lines (Figure 4). The known MSI pathways are noted by a red square and include the TGFβ, WNT, and MMR repair pathways. While most known drugs impact the RAS-MAPK signaling pathways, our analysis could highlight the potential for RIOK1 inhibition as a feasible therapeutic target for MSI-High CRC tumors (Figure 4). 

**Figure 5:**
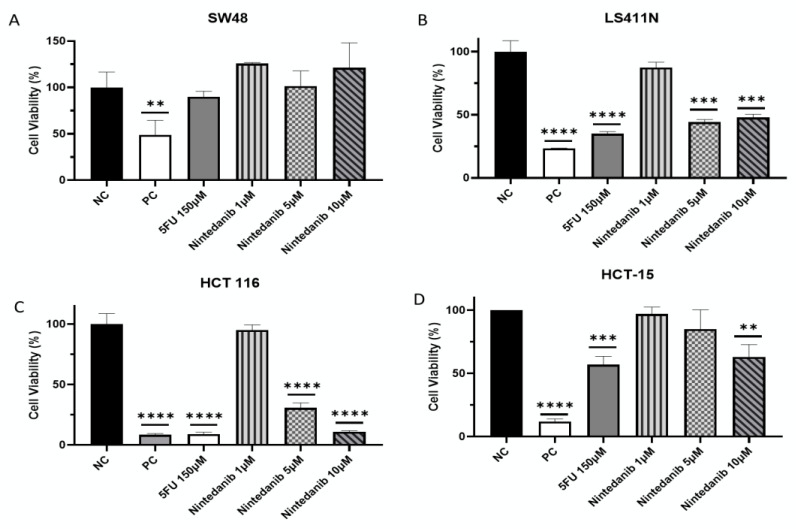
Cell growth inhibition in CRC MSI-High cell lines: Cell viability was measured using CCK-8 assay and is represented as the percentages of the untreated group value (NC). (**A**) Cytotoxic effect in SW48 treated with 150 µM 5FU or with different concentrations of Nintedanib for 48 h. Treatment with no additions was defined as NC (negative control) and treatment with 0.1% Triton X was defined as a PC (positive control). (**B**) Cytotoxic effect in LS411N cells and (**C**) HCT 116 cells treated with the same treatment for 72 h. (**D**) Cytotoxic effect in HCT-15 cells for 48 h. Data are expressed as the mean ± standard deviation of three independent experiments, performed in triplicate. ** *p* < 0.01 *** *p* < 0.001 and **** *p* < 0.0001 vs. the untreated NC group.

Both 5FU and Nintedanib showed p53-KRAS-dependent cell growth inhibition in MSI-High cell lines: Inhibitors of RIOK1 were tested in cell-growth assays to validate our computational predictions. These studies were conducted using SW48, a CRC MSI-High p53-KRAS-WT cell line, LS411N, a CRC MSI-High p53-Mut KRAS-WT cell line, HCT 116, a CRC MSI-High p53-WT KRAS-Mut cell line, and HCT-15, a CRC MSI-High p53-KRAS-Mut cell line (Table 3). The cell lines were treated with Nintedanib, a RIOK1 inhibitor [61], for 48 h or 72 h, and cell viability was monitored to determine effectiveness; 5FU, the most widely used chemotherapeutic agent for colorectal cancer, and Triton X-100 were used as positive controls [22]. The results showed no-to-very-limited sensitivity to both 5FU and Nintedanib in the SW48 cell line (p53-KRAS-WT) (Figure 5A, Table 3). In the LS411N cell line (p53-Mut, KRAS-WT), a significant increase in sensitivity to 150 µM 5FU (65% inhibition) and sensitivity to 5 and 10 µM Nintedanib (52% inhibition) was also observed (Figure 5B, Table 3). In the HCT 116 cell line (p53-WT, KRAS-Mut), the most significant inhibition was measured, with 89% inhibition of cell proliferation in both 5FU and 10 µM Nintedanib (Figure 5C, Table 3). Interestingly, HCT-15 (p53-KRAS-Mut) showed low sensitivity to Nintedanib (37%) and 5FU (43%) (Figure 5D, Table 3).

## 3. Materials and Methods

Identification of MSI-High and cell genotype in CRC cell lines: MSI-High cell lines were identified using data from (1) the ExPasy database (https://web.expasy.org/cgi-bin/cellosaurus/search (accessed on 2 September 2021)) [22,23]; (2) p53 mutation expression levels in colorectal cancer cell lines [26] and known MS status for these cell lines [29]. All data sources were cross-validated to assure a consensus definition of the MSI phenotype. A final list of eight MSI-High cell lines were identified, of which three had a p53 mutant genotype (LS411N-KRAS (WT), SNU-C2B-KRAS (p.G12D), and CCK-81-KRAS (WT)) and five had a wild-type p53 (WT) genotype (LS180-KRAS (p.G12D), HCT 116-KRAS (p.G13D), RKO-KRAS (WT), LoVo-KRAS (p.G13D; p.V14A), and SW48-KRAS (WT)). 

Pathway analysis: Pathways that were shown to be highly activated with relative activity > 0.85 were extracted from the ATLANTiC data set [29]. A cutoff of 0.85 was chosen as a threshold, as this provided a distinct profile between the two p53 subgroups. The data retrieved was assigned to either MSI and MSS but we elected to focus on MSI-High p53-WT or p53-Mut cell lines. The resulting highly activated pathways were clustered based on biological processes using the Gene Set Enrichment Analysis (GSEA DB) [30,31,32,33,34,35,36,37,38,39], PID [40], and BioCarta [41] databases. 

MSI-High Cell lines and culture conditions: SW48, LS411N, HCT 116, and HCT-15 cell lines were purchased from the American Type Culture Collection (ATCC, Rockville, MD, USA). SW48 cells were cultured in Leibovitz L-15 Medium, LS411N, and HCT-15 cells were cultured in RPMI-1640 Medium and HCT 116 cells were cultured in McCoy’s 5A Medium (ATCC). All media containing 10% fetal bovine serum (FBS), 100 units/mL penicillin, and 100 μg/mL streptomycin (Biological Industries Israel Beit-Haemek) at 37 °C. LS411N, HCT 116, and HCT-15 cells were cultured in a humidified atmosphere containing 5% CO_2_ while SW48 cells were cultured in a humidified atmosphere without CO_2_.

Cell Viability by WST-8 (Cell Counting Kit-8) Assay: The cytotoxicity was measured using a cell viability assay (Cell Counting Kit-8, Enzo Life Sciences, Inc., New York, NY, USA) following the manufacturer’s instructions. Briefly, 100 µL cells per well were seeded into 96-well plates (cells per well for SW48 and LS411N, 4300 cells per well for HCT 116, and 5000 cells per well for HCT-15) and incubated for 24 h. Cells were treated with 5FU (150 µM) or Nintedanib (1, 5, 10 µM) (*N* = 3, *n* = 6) and 0.1% Triton X was used as a positive control for cytotoxicity. Following 48 h or 72 h of incubation at 37 °C (48 h for SW48 and HCT-15 and 72 h for LS411N and HCT 116), WST-8 reagent was added to each well and the plate was placed in a 37 °C incubator for an additional 4 h. Optical density was measured at 450 nm on a BioTek microplate reader (Agilent Technologies, Inc., Santa Clara, CA, USA).

Drugs: Triton™ X-100 and 5FU were obtained from Merck (Darmstadt, Germany); 5FU was stored as 50 mg/mL stock in dimethyl sulfoxide (DMSO, Santa Cruz Biotechnology, Inc., Dallas, TX, USA) at −20 °C. Nintedanib was obtained from Angene International Limited (Nanjing Zhongshan Science and Technology Park, Nanjing, China) and stored as 25 mg/mL stock in DMSO at −20 °C.

Statistical analysis: Statistical analysis was performed using an unpaired t-test using GraphPad Prism version 9.1.2 for Windows, (San Diego, CA, USA). Significance calculations of the biological processes between MSI-High p53 WT vs. Mut were calculated using a chi-square test (SPSS version 20, IBM, Armonk, NY, USA).

## 4. Discussion

CRC is the third-most-common cancer in the world, arising from the colorectal epithelium as a result of the accumulation of genetic alterations. Two major mechanisms of genomic instability have been identified in sporadic CRC progression. The first, known as chromosomal instability (CIN), results from a series of genetic changes that involve the activation of oncogenes such as KRAS and inactivation of tumor suppressor genes such as p53, DCC/Smad4, and APC [63]. The second, known as microsatellite instability (MSI), results from the inactivation of the DNA mismatch repair genes such as MLH1/2/3, and the secondary mutation of genes with coding microsatellites, such as transforming growth factor receptor II (TGF-RII) and BAX [64]. MSI is detected in about 15% of all colorectal cancers, which has increased awareness of the diversity of colorectal cancers and has implications for the specialized management of patients [23].

It is now generally accepted that a few important intracellular signaling pathways, including Wnt/β-catenin signaling, Ras signaling, and p53 signaling, are frequently dysregulated in CRC. Likewise, CRC cell lines are known to possess a high frequency of TP53 (60%) mutations and to a lesser extent KRAS mutations (49%). Seeking to provide the largest benefit to the CRC MSI-High population, we wanted to identify SL partner(s) that serve as an Achilles heel specifically for the p53 cancer cell line. Originally, we did not include the KRAS genotype in our hypothesis because *KRAS*-mutated CRCs had a lower frequency in MSI-High CRC (15%, [13]). In addition, recent publications indicated that KRAS and p53 were mutually exclusive in KRAS CRC-altered tumors [17], so we initially focused on p53.

Dysregulation of the p53 tumor suppressor gene is one of the most frequent factors controlling the aggressive and metastatic features of CRC. Half of all colorectal cancers containing p53 mutations appear to be more chemo-resistant and have a poorer prognosis than those with wildtype (WT) p53 [10,11]. More importantly, patients with mutant p53 gene are often resistant to current therapies but there is emerging evidence from clinical trials that show that some small molecule inhibitors exert anticancer effects via reactivation and restoration of p53 function [65].

The rationale for clustering CRC cell lines based on their MSI-High phenotype is that these cell lines are known to exhibit clinical, pathological, and molecular characteristics which distinguish them from MSS cancer cell lines and p53 is a key regulator of cellular processes. Our analysis demonstrated that cell signaling, DNA repair, and immune system-related processes are significantly more abundant in MSI-High p53-Mut cell lines while cell-cycle checkpoint, metabolism of protein, metabolism of RNA, signal transduction, and WNT signaling were more pronounced in MSI-High p53-WT cell lines (Table 1). 

Pathway analysis demonstrated that four specific kinases were upregulated in these pathways: TBK1, CAMK1, CHUK, and RIOK1 (Table 2). Several reasons lead to the selection of RIOK1 as the target for further investigation, including that RIOK1 was found to be overexpressed in colon cancer cells, which promotes cell proliferation in vitro in human CRC [24,48]. In addition, RIOK1 was published to have an SL role in methylthioadenosine phosphorylase (MTAP) depleted cells [24,48]. In the context of p53, RIOK1 was shown to mediate p53 degradation and radioresistance in colorectal cancer [66]. Coincidentally, a recent study suggested that RIOK1 depletion is not generally toxic to cells but might represent an Achilles heel, specifically for RAS-driven cancers [67]. This was confirmed by another study that showed that RAS-driven tumor cells were dependent on RIOK1 activity of cell proliferation [24,68,69,70]. These recent findings justify our explorative work looking at RIOK1 inhibition in the context of MSI-High CRC population to determine if RIOK1 could create an SL phenotype in CRC MSI-High p53 cancers by targeting pathways downstream of the p53 cascade that is unique from the ones currently under clinical evaluation.

Treating MSI-High CRC cell lines bearing p53 mutations with Nintedanib (a known RIOK1 inhibitor) was expected to show differences in efficacy. This expectation was derived from the significant difference in treatment response and patient outcome based on their p53 genotype [12]. This observation, in addition to the diverse results observed in the in vitro cell cytotoxicity experiments, required the inclusion of additional information, e.g., KRAS genotype to our hypothesis and analysis. An analysis of drugs known to exhibit potency in MSI-High Mut p53 cell lines revealed that most drugs inhibit kinases belonging to pathways known to be aberrant in MSI cancer cells (e.g., MAPK, ERK, PI3K, TGFB, WNT, and mTOR signaling pathways (Figure 4)), while RIOK1-specific inhibition is novel. Characterization of RIOK in cancer using isogenic colon, breast, and lung cancer cell lines has shown that silencing of RIOK1 inhibited proliferation and invasiveness in 2D and 3D culture systems [24]. Depletion of RIOK1 in other cell lines affected the expression and phosphorylation of proteins involved in multiple biological processes, including ribosomal biogenesis, cell cycle progression, metabolic activity, NF-κB signaling, and metastases [24]. DepMap analysis showed that depletion of RIOK1 in MSI-High cell lines, especially in combination with a PMS1 KO, has a debilitating effect on cell viability and was recently noted as an anticancer CRC therapy [71]. In vitro studies showed varied cytotoxicity upon treatment with Nintedanib. Specifically, the SW48 cell line (p53 and KRAS double WT) showed complete resistance to both Nintedanib and 5FU with no effect on cell proliferation. 

CRC MSI-High cell lines bearing opposite genotypes of p53 and KRAS, where one gene is WT and the other mutant (e.g., LS411N, HCT 116) showed the highest sensitivity and the most significant reduction in cell proliferation (52% and 89%, respectively). Contradicting what we found, recent results demonstrated that RIOK1 shRNA treated the LoVo and HCT 116 cell lines (both MSI-High p53-WT and KRAS-Mut (G13D) showed no difference in cell apoptosis between untreated and RIOK1 shRNA-treated cell lines [66]. Of note, the findings that HCT 116, p53 WT, and KRAS-Mut-(G13D) showed the greatest sensitivity to the treatments could be explained by the observation in the clinics where patients with KRAS-G13D appeared to respond positively to drugs like cetuximab (used in treatment for metastatic colorectal cancer), suggesting this mutation is an exception to the rule that KRAS mutations are undruggable and confer resistance to drugs such as epidermal growth factor receptor (EGFR) inhibitors [72]. As noted, no toxicity was observed in SW48 cells (dual WT p53 and KRAS) when using Nintedanib. Surprisingly, the most modest resistance in cytotoxicity was observed in HCT-15 (dual p53 and KRAS Mut) which is opposite to the hypothesis of SL and MSI-High cell lines’ dependency on genes known to be essential for the cell’s wellbeing (e.g., p53 and KRAS). One possible explanation may have to do with the fact that HCT-15 bears a p53-(S241F) mutation which is characterized by p53 mutant gain of function (GOF) [73]. p53 GOF loses its tumor-suppressive functions and mediates oncogenic properties such as sustained proliferation; cell-death resistance, invasion, and metastasis; and tumor-promoting inflammation [74,75]. This variability in cytotoxicity is in line with a well-established phenomenon seen in CRC where responses to KRAS inhibitors are variable and the difference in treatment response in patients is based on their p53 genotype [12,76].

Large data sets of human genomic, transcriptional, proteomic, and epigenetic data are readily accessible from tumor cells, which allows increased analysis of cell-cycle pathways heterogeneity. Understanding these heterogeneities makes the development of personalized treatments targeting specific proteins possible. As a result, in 2020, the FDA approved Keytruda (pembrolizumab) for intravenous injection for the first-line treatment of patients with unresectable or MSI-High or mismatch repair deficient colorectal cancer. This marks the first immunotherapy approved for this patient population as a first-line treatment and which is administered to patients without also giving chemotherapy. This trend opens new opportunities for MSI-High patients regardless of the precise molecular mechanisms underlying the effect of RIOK1 inhibition. Our data support RIOK1 as a potential target for MSI-High subpopulation (with either p53 or KRAS Mut) tumor therapy that currently has no effective treatment [12]. RIO kinases exhibit ATPase activity and, as such, can potentially be targeted by small-molecule compounds. RIOK1 knockdown does not impair the proliferation of K562(MSS), Caco-2(MSS), and MCF-10A cells (MSS) and this suggests that RIOK1 depletion may not be generally toxic and, therefore, potentially suitable as an MSI-High targeted, specific therapeutic strategy [77]. 

Our results demonstrate that systems approaches can uncover nonobvious, mechanistic bases for clinical observations that otherwise defy expert-level explanation. These findings validate the methods we have been using to identify new targets/kinases for anticancer therapies [78]. RIOK1 represents a new anticancer drug target, and the chemical space of its inhibitors has just emerged, revealing exciting possibilities [24,79]. RIOK1 inhibition could serve as a single arm of combination therapy by exploiting the presence of targetable co-altered genes and we could enhance the anticancer efficacy in a tailored manner [80]. The outcome of such therapy will likely depend on the clinical genomics of the MSI-High cancer as pretreatment and upon acquired resistance to therapies [76].

## Figures and Tables

**Table 1:** Significantly differently activated biological processes found in MSI-High cell lines stratified by their p53 genotype. Highly activated pathways (using all 44 processes) were clustered into biological processes, followed by a chi-square test to identify significant, differently activated processes at each p53 subgroup. The *χ*^2^ value determines if the null hypothesis (no significant difference in process activation level) can be accepted/rejected while the *p*-value indicates the level of confidence. *N* represents the total number of highly activated pathways per cell line. *N* = 485 is the total number of highly activated pathways in MSI-High-WT p53 and *N* = 325 is the total number of highly activated pathways in MSI-High-Mut p53. Highlighted cells in the table represent the MSI-High subgroup where the biological processes were found to be significantly more activated with *N* ≥ 5.

Process Type	WT	MUT	*χ* ^2^	Significance
	% Within WT	% Within MUT		
	(*N* = 485)	(*N* = 325)		
Cell signaling	0.0 (*N* = 0)	1.5 (*N* = 5)	7.51	*p* < 0.01
Cell-cycle checkpoint	2.1 (*N* = 10)	0.6 (*N* = 2)	6.79	*p* < 0.01
DNA repair	1.8 (*N* = 9)	6.7 (*N* = 22)	14.4	*p* < 0.01
Immune system	13 (*N* = 63)	21.8 (*N* = 71)	11.06	*p* < 0.01
Metabolism of Proteins	3.7 (*N* = 18)	0.3 (*N* = 1)	9.84	*p* < 0.01
Metabolism of RNA	7.0 (*N* = 7)	3.7 (*N* = 12)	4	*p* < 0.05
Signal transduction	5.2 (*N* = 25)	1.5 (*N* = 5)	7.14	*p* < 0.01
WNT signaling	2.3 (*N* = 11)	0.0 (*N* = 0)	7.47	*p* < 0.01

**Table 2:** Kinase’s abundance filtered by their MS status and p53 genotype. Once we identified significantly different activation levels of biological processes, we searched for the most abundant kinases (using ATLANTiC’s portal and relative activity of >0.85). We identify four enriched kinases in MSI-High cell lines: TBK1 (belonging to foreign DNA and RNA/immune response signaling), RIOK1 (belonging to ribosome biogenesis in eukaryotes), CHUK (belonging to NF-Kβ signaling), and CAMK1 (belonging to calcium-triggered CaMKK-CaMK1 signaling cascade).

Cell Type	TBK1	RIOK1	CHUK	CAMK1
abundance (MSI-high-p53-Mut)	0.81	0.81	0.78	0.57
abundance (MSI-high-p53-WT)	0.85	0.85	0.91	0.86
abundance (MSS-p53-Mut)	0.61	0.69	0.58	0.58
abundance (MSS P53 WT)	0.68	0.61	0.66	0.71

**Table 3:** P53/KRAS status in the different CRC MSI-High cell lines and the toxic effect of Nintedanib (10 µM) on the cell lines.

Cell Line	p53 Status	KRAS Status	Toxicity
SW48	WT	WT	-
LS411N	Mut	WT	52%
HCT 116	WT	Mut	89%
HCT-15	Mut	Mut	37%

## Data Availability

Not applicable.

## Supplementary Materials

The following supporting information can be downloaded at: https://www.mdpi.com/article/10.3390/molecules28114452/s1, Figure S1: RIOK1 expression levels in CRC cell lines stratified by their MS status; Table S1: Identification of the most abundant kinases that could account for the identified highly activated pathways [81,82].
