# Supplementary material for: Riok1, A Novel Potential Target in MSI-High p53 Mutant Colorectal Cancer Cells
Source: Molecules. 2023 May 31;28(11):4452. doi: 10.3390/molecules28114452 (PMC10254355; doi:10.3390/molecules28114452)
Supplement: Supplementary file 1 [file molecules-28-04452-s001.zip › molecules-2389486-supplementary.pdf]

Table S1: Identification of the most abundant kinases that could account for the identified highly activated pathways. To conduct this analysis, we used the kinase abundance data per MSI/MSS and p53 WT/MUT reported at the ATLANTiC's portal to identify eight kinases that could explain the pathway activation pattern. To better understand the dark kinases identified and their function in human biology we relayed on Dark Kinase Knowledgebase (https://darkkinome.org/ (accessed on 2 September 2021)) [81] as well as Integrated Network and Dynamical Reasoning Assembler (INDRA) which was used to draw pathway information for these kinases [82].

Kinase by abundance

MSI-High-p53-Mut	TBK1	MAPK12	MAP2K3	RIOK1	MAP2K1	CAMK1	CHUK	TGFBR1
SNU-C2B	0.77	0.67	0.78	0.81	0.76	0.32	0.88	0.75
LS411	0.86	0.73	0.78	NA	0.84	0.61	0.62	0.81
CCK-81	0.8	0.72	0.75	NA	0.85	0.78	0.84	0.77
**Average (MSI-High-p53-Mut)**	0.81	0.71	0.77	0.81	0.82	0.57	0.78	0.78
**Std**	0.05	0.03	0.02	0.00	0.05	0.23	0.14	0.03
**MSI-High-p53-WT**	**TBK1**	**MAPK12**	**MAP2K3**	**RIOK1**	**MAP2K1**	**CAMK1**	**CHUK**	**TGFBR1**
LS180	0.89	NA	NA	0.89	0.85	0.85	NA	0.34
LOVO	0.86	0.95	NA	NA	0.84	NA	0.91	0.73
HCT116	0.89	0.34	0.73	0.86	0.81	0.8	0.85	0.73
RKO	0.82	0.76	0.85	0.8	NA	0.98	NA	0.65
SW48	0.82	0.95	0.83	0.87	0.6	0.79	0.99	0.7
**Average (MSI-High-p53-WT)**	0.86	0.75	0.80	0.86	0.78	0.86	0.92	0.63
**Std**	0.04	0.29	0.06	0.04	0.12	0.09	0.07	0.17
**MSS-p53-Mut**	**TBK1**	**MAPK12**	**MAP2K3**	**RIOK1**	**MAP2K1**	**CAMK1**	**CHUK**	**TGFBR1**
NCI-H716	NA	0.43	0.41	0.52	NA	0.42	NA	NA
SW948	0.65	0.35	0.77	0.82	0.9	0.67	0.68	0.81
HCA-46	0.6	0.29	NA	NA	0.6	0.58	0.56	0.58
SW403	0.65	NA	0.78	0.82	0.88	0.77	0.76	0.75
T84	0.61	NA	NA	NA	0.61	0.38	0.32	NA
CoCM-1	NA	0.43	0.52	NA	NA	NA	NA	NA
CACO2	0.56	0.81	0.69	0.63	0.35	0.64	0.59	NA
**Average (MSS-p53-Mut)**	0.61	0.46	0.63	0.70	0.67	0.58	0.58	0.71
**Std**	0.04	0.20	0.16	0.15	0.23	0.15	0.17	0.12
**MSS-p53-WT**	**TBK1**	**MAPK12**	**MAP2K3**	**RIOK1**	**MAP2K1**	**CAMK1**	**CHUK**	**TGFBR1**
C32	0.64	0.68	0.75	0.66	NA	0.82	NA	0.72
CaR-1	0.64	0.68	0.8	0.77	0.9	0.73	0.67	0.8
Colo-678	0.73	0.73	0.84	0.35	NA	0.71	NA	0.83

LS123	0.6	0.64	0.75	0.7	0.73	0.68	0.63	0.75
C106	0.73	NA	NA	0.39	0.76	0.44	NA	0.7
C75	NA	NA	NA	NA	0.56	0.74	NA	0.67
C99	0.89	NA	NA	NA	0.67	0.88	0.35	NA
HT55	0.79	NA	NA	NA	0.75	0.68	0.69	0.7
LS513	0.72	NA	NA	0.84	0.93	0.78	0.78	0.88
SW1417	0.55	0.6	0.25	NA	NA	0.46	0.26	0.66
SW1116	0.77	NA	NA	NA	0.7	NA	0.74	NA
LIM1863	0.8	NA	NA	0.36	0.82	NA	0.73	NA
C80	0.71	NA	0.47	NA	0.81	0.84	0.8	0.44
RCM-1	0.81	NA	NA	0.75	0.84	NA	0.74	0.5
C125-PM	0.37	NA	NA	NA	0.67	0.86	0.7	NA
C70	0.77	NA	NA	NA	0.68	0.87	0.83	NA
Colo 320DM	NA	0.73	0.84	0.35	NA	0.71	NA	0.83
SW480	0.35	0.74	0.76	0.83	NA	0.85	NA	0.82
Colo 741	0.66	0.71	0.7	0.68	NA	0.29	0.6	NA
**Average (MSS P53 WT)**	0.68	0.69	0.68	0.61	0.76	0.71	0.66	0.72
**Std**	0.15	0.05	0.20	0.20	0.10	0.17	0.17	0.13

Shechter et al

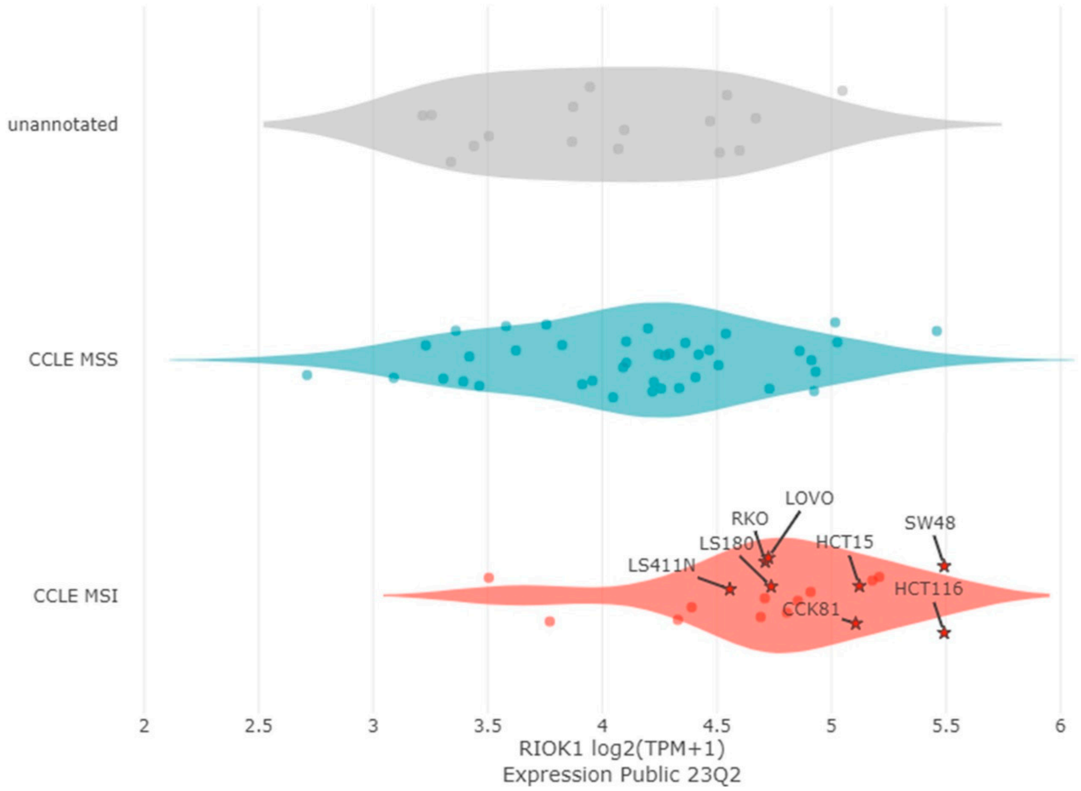

Figure S1: RIOK1 expression levels in CRC cell lines stratified by their MS status. From the eight cell lines whose genetic profile and MS status was confirmed, seven were found in the DepMap portal (Broad Institute) and appear in the plot. The cell lines annotated in blue circle are MSI-high p53 Mut. TPM stands for transcript per million, and the sum of all TPM values is the same in all samples, such that a TPM value represents a relative expression level that, in principle, should be comparable between samples.
